# To Assess if Prescribing Psychotropic Medication for Patients With Personality Disorder Diagnosis in the Absence of Any Other Psychiatric Comorbidities, Improves the Clinical Outcome

**DOI:** 10.1192/bjo.2026.11950

**Published:** 2026-06-30

**Authors:** Tanzida Haque, Kirti Shastri, Eze Blessing, Victoria Jones, Bassem Naguib

**Affiliations:** Lancashire and South Cumbria NHS Foundation Trust, Ormskirk, Liverpool, United Kingdom

## Abstract

**Aims:**

According to NICE guideline, psychotropic medication has limited role to improve clinical outcome in EUPD patients. However, the practice of utilising psychotropic medication is increasing. The aim of this audit is to assess whether prescribing psychotropic medication in the absence of any other psychiatric co morbidities improves the clinical outcome.

**Methods:**

The audit was completed at the Scarisbrick In-patient unit at Lancashire and South Cumbria NHS Foundation Trust. The number of hospital admission within next 1 year of starting psychotropic medication was used as the indicator for clinical outcome. Data was collected retrospectively from the electronic system from the patients’ cohort who were admitted from 01/2023-12/2023, with diagnosis of EUPD in the absence of other psychiatric comorbidities, (N=29) to assess if prescribing psychotropic medication can reduce number of hospital admission within next 1 year (between 01/24- 12/2024).

**Results:**

The result shows high rate of psychotropic medication used in patients with EUPD diagnosis in the absence of any other psychiatric comorbidities. 62% patients were already on antidepressants and 55% on antipsychotics prior to admission. After medication review post admission, 55% started on psychotropics, including 41% on antipsychotics, 35% on antidepressants, and 14% on mood stabilisers.

Despite being on psychotropic medications, the readmission rate within next 1 year remains high. 58.62 % (N=17) patients had re-admissions within one year of starting psychotropics. Of those, 71% (N=12)had more than one admission. 65% (N=11) of readmissions were under the Mental Health Act. For 76% (N=13) patients, admission lasted more than 2 weeks and 24% had crisis admission.

The audit focuses solely on pharmacological data. There is no mention of psychological input, crisis plans, care coordination, which is central to NICE/RCPsych recommendations.

**Conclusion:**

High proportion of patients with EUPD are prescribed psychotropic medications before and during admission, despite the limited evidence supporting their effectiveness in this population. This is not consistent with NICE, which cautions againstroutine psychotropic use in EUPD in the absence of comorbidities or clear indications. Over half of patients were re-admitted to hospital within next one year of starting psychotropic medication and most had multiple re-admissions. The high re-admission rate, particularly under the MHA and with prolonged stays, raises concern about the effectiveness of psychotropic medication in reducing relapse or improving long-term outcomes in EUPD. The data appears to support NICE guidance that psychotropic medications should not be used routinely in the absence of comorbid mental illness, as their benefit in preventing relapse is questionable.

